# Evaluation of Immature Platelet Fraction in Lower Respiratory Tract Infections: A Retrospective Study

**DOI:** 10.7759/cureus.9227

**Published:** 2020-07-16

**Authors:** Vasiliki E Georgakopoulou, Dimitrios Mermigkis, Konstantinos Mantzouranis, Christos Damaskos, Despoina Melemeni, Eleni A Alafaki, Georgios Petsinis, Nikolaos Garmpis, Evgenia Karakou, Anna Garmpi, Agathi Lekkakou, Pagona Sklapani, Nikolaos Trakas, Rea Chatzikyriakou, Xanthi Tsiafaki

**Affiliations:** 1 Pulmonology Department, Laiko General Hospital, Athens, GRC; 2 1st Pulmonology Department, Sismanogleio Hospital, Athens, GRC; 3 Renal Transplantation Unit, Laiko General Hospital, Athens, GRC; 4 N.S. Christeas Laboratory of Experimental Surgery and Surgical Research, Medical School, National and Kapodistrian University of Athens, Athens, GRC; 5 Hematology, Sismanogleio Hospital, Athens, GRC; 6 Second Department of Propedeutic Surgery, Laiko General Hospital, Medical School, National and Kapodistrian University of Athens, Athens, GRC; 7 Biochemistry Department, Sismanogleio Hospital, Athens, GRC; 8 First Department of Propedeutic Internal Medicine, Laiko General Hospital, Medical School, National and Kapodistrian University of Athens, Athens, GRC; 9 Cytopathology Department, Mitera Hospital, Athens, GRC

**Keywords:** platelets, respiratory infections, immature platelet fraction

## Abstract

Introduction

Immature platelet fraction (IPF) is a parameter of an automated hematologic analyzer and is related to platelet size and cytoplasmic RNA content. It reflects thrombopoiesis and is often used as the marker of platelet activity. IPF has been evaluated mostly in hematologic disorders and has also been evaluated in patients with gestational hypertension, sepsis, autoimmune diseases and in hospitalised patients with neutrophilia. Platelets, asides from the maintenance of hemostasis, release inflammatory mediators that can modify leukocyte and endothelial responses to various inflammatory stimuli. Lower respiratory tract infections are the leading cause of death from infections worldwide. The role of platelets in lower respiratory tract infections has been reported in many studies. IPF, which is related to platelet activation, has not been evaluated in patients with lower respiratory tract infections.

Methods

The study involved patients who fulfilled the criteria of community-acquired pneumonia (CAP) and aspiration pneumonia (AP). In addition, age and sex-matched healthy controls were involved. Whole blood samples were collected from healthy controls and from the patients on admission. The mean IPF% and C-reactive protein (CRP) levels were measured in patients with CAP, in patients with AP and in healthy controls. The mean IPF% values in patients with infection were compared to mean IPF% values in healthy controls. The mean IPF% values were compared to mean CRP levels in patients with infection. Additionally, the mean IPF% values in patients that died in the first 14 days were compared to the mean IPF% values in patients that were alive. The statistical analysis of data was performed with the Statistical Package for the Social Sciences (SPSS) for Windows, Version 13.0 (SPSS Inc, Chicago, IL).

Results

The study population consisted of 45 patients (27 patients with CAP and 18 patients with AP), 27 males and 18 females, with a mean age of 72.11 ± 16.4 years and 39 healthy controls, 22 males and 17 females with a mean age of 64.2 ± 14.8 years. The mean CRP levels in patients with infection were 155.2±119.1 mg/dl. The mean IPF% value of patients with infection was 2.76 ± 2.27 and the mean IPF% value of controls was 1.72 ± 0.77 (p < 0.006). The IPF% value in patients with CAP was 2.55 ± 2.02 and in patients with AP 3.07 ± 2.64 (p = 0.595). The mean IPF% value in patients with infection had no linear correlation with CRP value in these patients (r = 0.076, p = 0.62). The mean IPF% value in all patients that died in the first 14 days was 3.75 ± 2.44 and the mean IPF% value in all patients alive was 2.35 ± 2.11 (p = 0.06). The mean IPF% value in patients with CAP who died in the first 14 days of hospitalisation was 5.54 ± 3.17 and in patients with CAP who were alive was 1.87 ± 0.72 (p = 0.06). The mean IPF% value in patients with AP who died was 2.63 ± 0.85 and in patients with AP who were alive was 3.41 ± 3.51 (p = 0.554).

Conclusions

Mean IPF% value is greater in patients with lower respiratory tract infections, including CAP and AP, compared to healthy controls. There is no linear correlation between IPF values and CRP values in patients with lower respiratory tract infections. In addition, there is a difference in mean IPF% value between patients who died in the first 14 days of hospitalisation compared to those who were alive, but not statistically significant.

## Introduction

Immature platelet fraction (IPF) represents the percentage of platelets in circulation retaining RNA. It is an innovative parameter measuring reticulated platelets and is considered a rapid automated biomarker for differentiating cases of thrombocytopenia. It is calculated by flow cytometry or hematology analysers [1]. High IPF values are observed in conditions characterised by rapid platelet destruction and low IPF values are observed in disorders with low bone marrow activity [2].

IPF reference intervals are not clear. In a study in a large Danish population, the reference interval for IPF was 1.3%-9% and in another study in American population was reported that reference intervals for IPF were 0.5%-3.2% in men and 0.4%-3.0% in women [3,4].

IPF has been evaluated in numerous diseases. IPF has been evaluated in hematologic disorders such as immune thrombocytopenic purpura in adults [5] and childhood immune thrombocytopenia [6]. IPF levels have been found significantly higher in patients with gestational hypertension compared to controls [7]. IPF has been reported to have a comparable efficiency to C-reactive protein (CRP) in discriminating patients who had sepsis and those who had not and to be predicting the risk of developing sepsis in patients admitted to intensive care unit [8]. IPF could be used as a screening tool for bacterial infection in hospitalised patients with neutrophilia with significant correlation to blood culture positivity [9]. In addition, the role of IPF as a biomarker in patients with autoimmune diseases has been reviewed [1].

Platelets have a major role in the maintenance of hemostasis. However, they release several inflammatory mediators, not related to hemostasis. These mediators modify leukocyte and endothelial responses to various inflammatory stimuli. Platelets interact with neutrophlis, lymphocytes, monocytes and endothelial cells and therefore have a significant role in inflammation and immune responses [10].

Platelets contain several organelles such as mitochondria, lysosomes and peroxisomes, and numerous intracellular immune mediators stored in granules and vesicles. In addition, platelets express considerable membrane receptors and contain cytoplasmic mRNA, which can synthesize a few proteins. These receptors and proteins allow platelets to interact with leukocytes and endothelial cells. Thus, platelets can regulate immune responses at the sites of platelet activation systemically. Platelet receptors and the molecules stored in platelet granules regulate platelets functions. There are three main types of platelet granules: α-granules, dense granules and lysosomal granules. α-granules are the most abundant and largest granules and they contain close to 300 proteins, including chemokines, such as P-selectin, CXCL1, CXCL5, CXCL7, CXCL12, macrophage inflammatory protein (MIP)-1α, regulated on activation normal T expressed and secreted (RANTES) and platelet factor 4 (PF4). Dense granules are smaller, less abundant and store small molecules, such as ADP, ATP, inorganic polyphosphate, histamine and serotonin, while lysosomal granules are scattered and contain proteases and glycosidases [11].

When platelets are stimulated, granules undergo exocytosis and release their molecules with immunomodulating properties into the extracellular space. In addition, molecules found on the inner granule membrane become surface-expressed [11]. Activated platelets also release interleukin-1β (IL-1β), which is not granule-stored but produced upon platelet activation [12].

Lower respiratory tract infections are the leading cause of death from infections worldwide [13]. The role of platelets in lower respiratory tract infections has been reported in many studies. The activation of platelets by the S.pneumoniae, the most common cause of community-acquired pneumonia (CAP), in vitro, has been reported [14]. The mechanisms of platelet activation by the pneumococcus and the role of platelets in CAP have been described [15]. It has been reported that the presence of phage protein pblB on the pneumococcal chromosome probably results to increased mortality in patients with an invasive S. pneumoniae infection, which may be explained by enhanced platelet activation [16].

Mean platelet volume (MPV), which is another parameter related to platelet activation, has been evaluated in lower respiratory tract infections [17]. Elevated MPV strongly predicts in-hospital and long-term mortality in patients hospitalised for CAP, while changes in MPV during intensive care unit admission can probably be used as a prognostic index of mortality in intensive care unit patients with pneumonia [18,19].

However, IPF, which also is related to platelet activation, has not been evaluated in patients with lower respiratory tract infections. We aim to evaluate IPF in patients with lower respiratory tract infections.

## Materials and methods

The study involved patients who fulfilled the criteria of CAP and aspiration pneumonia (AP). The exclusion criteria were: patients <18 years old, patients with a history of solid tumors or hematological malignancy, patients suffering from disease or receiving therapy that suppresses the bone marrow activity, patients receiving antiplatelet therapy and patients with platelet counts of less than 150 x10³/μl. In addition, age and sex-matched healthy controls without infection, and with platelet counts of more than 150 x10³/μl, were involved. Whole blood samples were collected from healthy controls and from the patients on admission. The mean IPF% and CRP levels were measured in patients with CAP, in patients with AP and in healthy controls. IPF was measured in automated Sysmex ΧΕ 2100 hematology analyzer. Serum levels of CRP were determined by immunoturbidimetric assay on Roche Cobas 6000 c501 analyzer. Reference range for IPF% in our laboratory is 1%-5% and normal values for CRP are values less than 6 mg/dl. The mean IPF% values in patients with lower respiratory infection were compared to mean IPF% values in healthy controls. The IPF% values were compared to CRP levels in patients with infection. Additionally, the mean IPF% values in patients who died in the first 14 days of hospitalisation were compared to the IPF% values in patients that were alive. The statistical analysis of data was performed with the Statistical Package for the Social Sciences (SPSS) for Windows, Version 13.0 (SPSS Inc., Chicago, IL). Continuous variables were tested for normality of distribution by the Kolmogorov-Smirnov test. For normally distributed values, descriptive results are presented as mean (standard deviation). The statistical control for normal values performed with: (a) independent samples T test for normally distributed data, (b) one-way ANOVA and (c) Pearson x^2^ for control of independence of variables. All p-values were two-sided and 5% was chosen as the level of statistical significance.

## Results

The study population consisted of 45 patients (27 patients with CAP and 18 patients with AP), 27 males and 18 females, with a mean age of 72.11 ± 16.4 years and 39 healthy controls, 22 males and 17 females with a mean age of 64.2 ± 14.8 years (Table 1).

**Table 1:** Characteristics of the study population C, controls; AP, aspiration pneumonia; CAP, community-acquired pneumonia; IPF, immature platelet fraction. Status: alive/dead in the first 14 days of hospitalisation

	C, n = 39	AP, n = 18	CAP, n = 27	C versus AP	C versus CAP	AP versus CAP
Gender (males/females)	22/17	11/7	16/11	0.738	0.818	0.901
Age(years) (SD)	64.2 (14.8)	77.5 (13.3)	69.7 (17.1)	<0.01	0.339	0.238
CRP (mg/dl) (SD)	3.28 (1.05)	188.3 (115.9)	133.2 (118.1)	<0.0001	<0.0001	0.092
IPF (%) (SD)	1.72 (0.77)	3.07 (2.64)	2.55 (2.02)	<0.023	0.148	0.595
Status (alive/dead)	39/0	10/8	22/5	<0.0001	<0.0001	0.060

**Figure 1:**
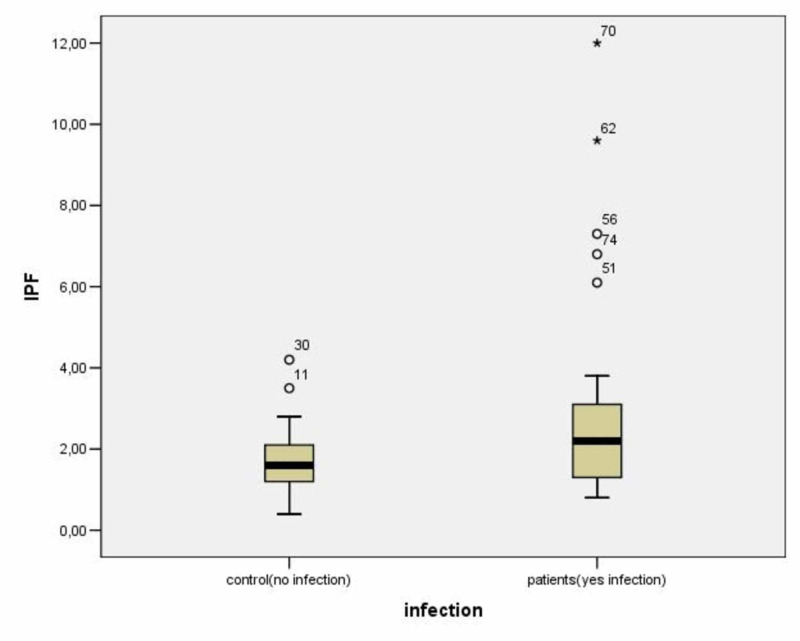
Comparison of IPF% values between infected and healthy controls 11, 30, 51, 56, 74: outliers; 62, 70: extreme values. IPF, immature platelet fraction

The mean CRP levels in patients with lower respiratory tract infections were 155.2 ± 119.1 mg/dl (Table 2). The mean IPF% value of patients with lower respiratory tract infections was 2.76 ± 2.27 and the mean IPF% value of controls was 1.72 ± 0.77 (p < 0.006) (Table 2; Figure 1).

**Table 2:** Mean IPF% and CRP values in patients with infection and healthy controls C, controls; IPF, immature platelet fraction; CRP, C-reactive protein

	C, n = 39	Patients, n = 45	C versus patients
IPF (%) (SD)	1.72(0.77)	2.76(2.27)	<0.006
CRP (mg/dl) (SD)	3.28(1.05)	155.2(119.1)	<0.0001

**Figure 2:**
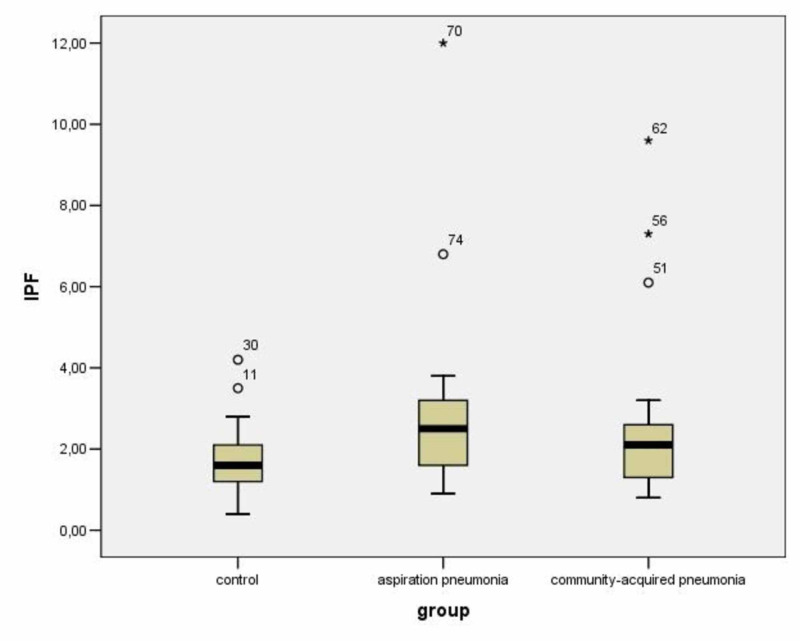
Comparison of IPF% values between groups 11, 30, 51, 56, 74: outliers; 62, 70: extreme values. IPF, immature platelet fraction

**Figure 3:**
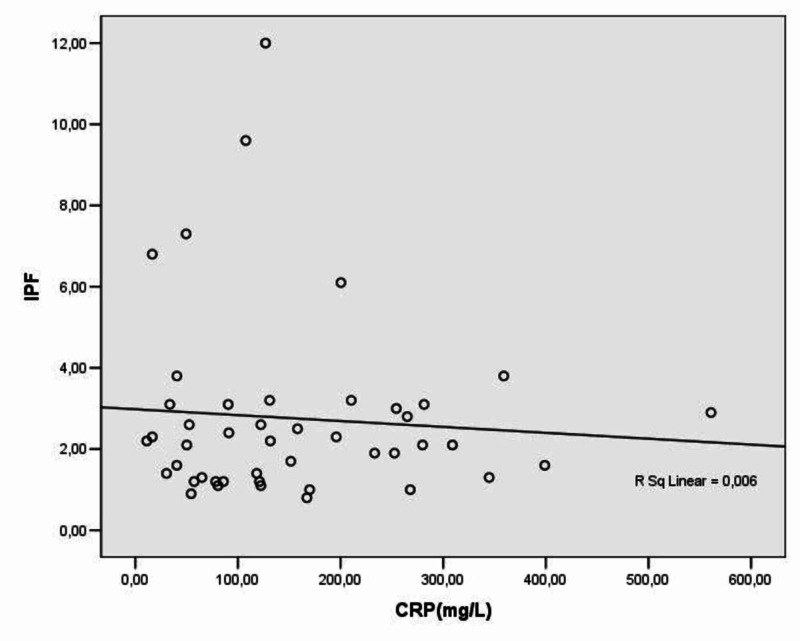
Correlation between IPF% and CRP values in infected patients. CRP, C-reactive protein; IPF, immature platelet fraction

**Figure 4:**
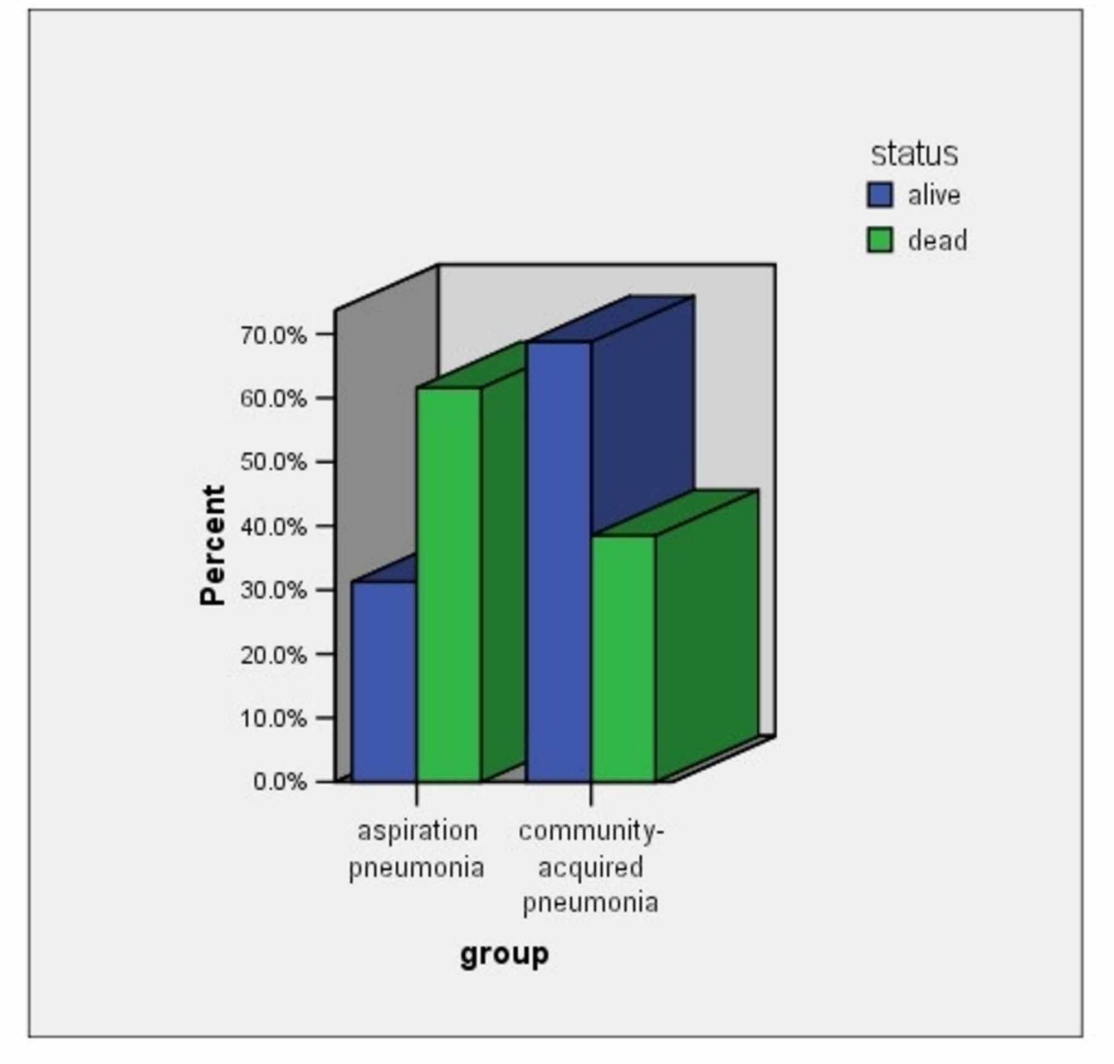
People alive/dead in each group of infection in the first 14 days of hospitalisation

**Figure 5:**
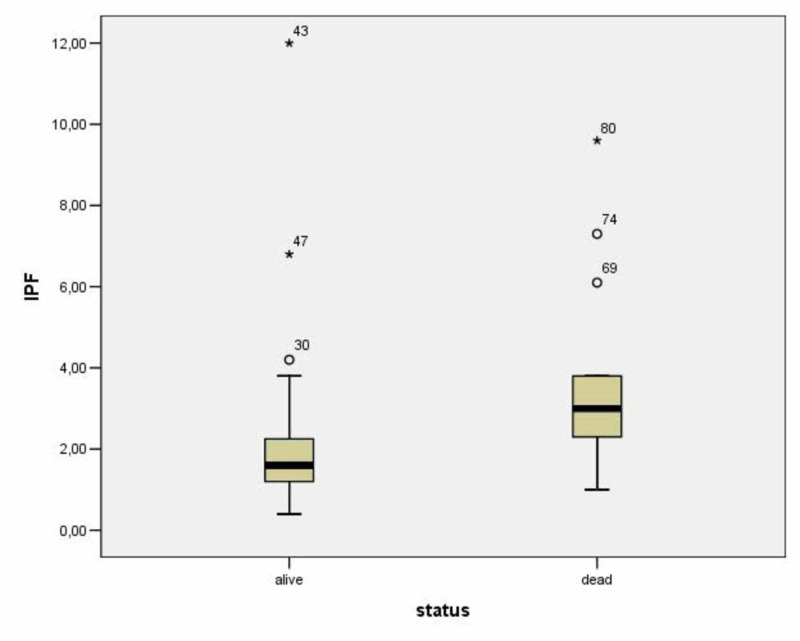
Comparison of IPF% values between dead and alive patients in the first 14 days 30, 69, 74: outliers; 43, 47, 80: extreme values; IRF, immature platelet fraction

The mean IPF% value in patients with CAP was 2.55 ± 2.02 and in patients with AP 3.07 ± 2.64 (p = 0.595) (Figure 2). The IPF% values in patients with lower respiratory tract infections had no linear correlation with CRP values in these patients (r = 0.076, p = 0.62). (Table 3; Figure 3). Thirteen patients died in the first 14 days of hospitalisation [eight patients (61.5%) with AP and five patients (18.5%) with CAP (Figure 4). The mean IPF% value in all patients that died in the first 14 days was 3.75 ± 2.44 and the mean IPF% value in all patients that were alive was 2.35 ± 2.11 (p = 0.06) (Table 4; Figure 5). The mean IPF% value in patients with CAP who died in the first 14 days of hospitalisation was 5.54 ± 3.17 and in patients with CAP who were alive was 1.87 ± 0.72 (p = 0.06). The mean IPF% value in patients with AP who died in the first 14 days of hospitalisation was 2.63±0.85 and in patients with AP who were alive was 3.41 ± 3.51 (p = 0.554) (Table 5).

**Table 3:** Correlation between IPF% values and CRP values in patients with lower respiratory tract infections CRP, C-reactive protein; IPF, immature platelet fraction

REGRESSION	r	r^2^	p
CRP (mg/dl) = 2.981-0.001 (IPF (%))	0.076	0.006	0.620

**Table 4:** Mean IPF% values in dead and alive patients in the first 14 days of hospitalisation CRP, C-reactive protein; IPF, immature platelet fraction

Status	Alive, n =32	Dead, n = 13	Alive versus Dead
IPF (%) (SD)	2.35 (2.11)	3.75 (2.44)	0.06
CRP (mg/dl) (SD)	141.8 (124.2)	188.3 (102.3)	0.239

 

**Table 5:** Mean IPF% values and mean CRP values in patients with AP and CAP, dead and alive in the first 14 days of hospitalisation AP, aspiration pneumonia; CAP, community-acquired pneumonia; CRP, C-reactive protein; IPF, immature platelet fraction

	Status	Alive, n = 10	Dead, n = 8	Alive versus Dead
AP	IPF (%) (SD)	3.41(3.51)	2.63(0.85)	0.554
	CRP (mg/dl) (SD)	158.6 (119.9)	225.5 (106.3)	0.235
	Status	Alive n = 22	Dead n = 5	Alive versus Dead
CAP	IPF (%) (SD)	1.87 (0.72)	5.54 (3.17)	0.06
	CRP (mg/dl) (SD)	134.1(128.2)	128.9(66.7)	0.931

## Discussion

According to our results, there was a difference in mean IPF% value between patients with AP compared to the patients with CAP but no statistically significant. However, there was a statistically significant difference in mean IPF% value between all patients with respiratory infections and healthy controls, indicating increased bone marrow platelet production in infected patients. Platelets are anucleate blood cells, with intimate relationship with the lungs, which transit the pulmonary vessels and are present in alveolar capillaries. Megakaryocytes, the cells that are the precursors of platelets, are present in the mammalian lungs. There is evidence that the lungs are sites of thrombopoiesis, and that human lungs are reservoirs for platelets and release them in response to specific stimuli [20]. In addition, there is evidence that platelets regulate vascular permeability in the lungs and the barrier function of the alveolar capillaries. Recent studies indicate that platelets have a key role in pulmonary immune responses and integrity but that these cells can also contribute to injury in lung diseases [20].

As mentioned in the introduction, the platelet activation related to S. pneumoniae has been studied [14]. In addition, platelet activation associated with Staphylococcus aureus and Klebsiella pneumoniae-induced sepsis has been studied in numerous animal models of pulmonary bacterial infections [21]. There is evidence that platelets have an important role in the immune response to respiratory bacterial infection with P. aeruginosa. It has been reported that experimentally-induced platelet depletion leads to increased pulmonary bacterial growth and systemic bacterial dissemination indicating that platelets play a significant role in preventing bacterial lung infections [22]. Besides bacterial infections, the role of platelets in lower respiratory tract viral infections has been reported. Schrottmaier et al. described the interaction between platelets and leukocytes and the subsequent alteration in cytokine production as a possible mechanism involved in defense against the respiratory syncytial virus (RSV) [23].

CRP was identified in 1930 and is considered to be an acute-phase protein, indicating infectious or inflammatory disorders [24]. CRP has been studied as a diagnostic tool and as a biomarker for disease severity in lower tract infections and as a marker for prognosis in patients with CAP [25-27]. However, in our study, the IPF% values in patients with lower respiratory tract infections had no linear correlation with CRP values in these patients (r = 0.076, p = 0.62).

Liu et al. studied the correlation between the percentage of immature platelets and infection in 190 patients with a body temperature > 37.3°C or < 36°C and suspicious of infection, who were hospitalized and concluded that the sensitivity and specificity for diagnosing infection were, respectively, 91.78% and 93.18% when IPF% and CRP were used in combination [28]. IPF% changed dynamically during the course of the infection and recovered to lower than 5.5% at two to seven days before the body temperature was normal.

In our study, there was a difference in mean IPF% value between patients that were alive and patients who died in the first 14 days of hospitalisation, with greater mean IPF % value noticed among patients that died in the first 14 days of hospitalisation, but not statistically significant. These results indicate a greater platelet bone marrow production in patients who died. The association between platelet counts and mortality in patients with lower tract infections has been reported. Mirsaeidi et al. in their retrospective cohort study concluded that platelet abnormalities are associated with mortality in patients hospitalized with CAP and evaluating an initial complete blood count test in patients with CAP, an abnormal platelet count is a better predictor of outcome than an abnormal leukocyte count [29]. Brogly et al. in their multicentre observational study reported that thrombocytopenia is an independent predictor of mortality in patients with severe CAP [30].

The study has some limitations. The fact that all the patients included in this study were enrolled in a single medical center and the relatively small sample size of patients lead to limitations in the generalizability of the results. A further well-controlled, prospective study is needed to clarify the relationship between IPF and lower respiratory tract infections.

## Conclusions

Mean IPF% values are greater in patients with lower respiratory tract infections, including CAP and AP, compared to healthy controls. There is no linear correlation between IPF values and CRP values in patients with lower respiratory tract infections. In addition, there is no statistically significant difference in mean IPF% value between patients that died in the first 14 days of hospitalisation compared to those who were alive. Based on our study, we cannot conclude that IPF is a reliable inflammatory marker or prognostic factor for lower respiratory tract infections. To our knowledge, the current study is the first to evaluate IPF in patients with lower respiratory tract infections. Further studies are needed to establish the role of IPF as a biomarker in these patients.
